# Gingival shape analysis using surface curvature estimation of the intraoral scans

**DOI:** 10.1186/s12903-022-02322-y

**Published:** 2022-07-12

**Authors:** Marko Kuralt, Alja Cmok Kučič, Rok Gašperšič, Jan Grošelj, Marjeta Knez, Aleš Fidler

**Affiliations:** 1 Department of Restorative Dentistry and Endodontics, University Medical Centre Ljubljana, Hrvatski trg 6, 1000 Ljubljana, Slovenia; 2 Faculty of Medicine, University of Ljubljana, Ljubljana, Slovenia; 3 Public Health Centre Celje, Celje, Slovenia; 4 Department of Oral Medicine and Periodontology, University Medical Centre Ljubljana, Ljubljana, Slovenia; 5 Department of Oral Medicine and Periodontology, Faculty of Medicine, University of Ljubljana, Ljubljana, Slovenia; 6 Faculty of Mathematics and Physics, University of Ljubljana, Ljubljana, Slovenia; 7 Department of Endodontics and Operative Dentistry, Faculty of Medicine, University of Ljubljana, Ljubljana, Slovenia

**Keywords:** Gingivitis, Periodontitis, Computer-assisted image analysis, Dental model, Shape analysis, Curvature, Optical scanning

## Abstract

**Background:**

Despite many advances in dentistry, no objective and quantitative method is available to evaluate gingival shape. The surface curvature of the optical scans represents an unexploited possibility. The present study aimed to test surface curvature estimation of intraoral scans for objective evaluation of gingival shape.

**Methods:**

The method consists of four main steps, i.e., optical scanning, surface curvature estimation, region of interest (ROI) definition, and gingival shape analysis. Six different curvature measures and three different diameters were tested for surface curvature estimation on central (n = 78) and interdental ROI (n = 88) of patients with advanced periodontitis to quantify gingiva with a novel gingival shape parameter (GS). The reproducibility was evaluated by repeating the method on two consecutive intraoral scans obtained with a scan-rescan process of the same patient at the same time point (n = 8).

**Results:**

Minimum and mean curvature measures computed at 2 mm diameter seem optimal GS to quantify shape at central and interdental ROI, respectively. The mean (and standard deviation) of the GS was 0.33 ± 0.07 and 0.19 ± 0.09 for central ROI using minimum, and interdental ROI using mean curvature measure, respectively, computed at a diameter of 2 mm. The method’s reproducibility evaluated on scan-rescan models for the above-mentioned ROI and curvature measures was 0.02 and 0.01, respectively.

**Conclusions:**

Surface curvature estimation of the intraoral optical scans presents a precise and highly reproducible method for the objective gingival shape quantification enabling the detection of subtle changes. A careful selection of parameters for surface curvature estimation and curvature measures is required.

**Supplementary Information:**

The online version contains supplementary material available at 10.1186/s12903-022-02322-y.

## Background

Detection and diagnosis of gingival and periodontal conditions is a complex process requiring knowledge, experience, and skills [1]. First, gingival tissues are assessed for the presence or absence of inflammation by assessing the tissues’ redness and degree of swelling descriptively or as part of various qualitative or semi-quantitative indices [2–4]. Further examination also includes an invasive but more objective component evaluating the tissues’ tendency to bleed upon provocation using the periodontal probe in a dichotomous or semi-quantitative manner [1, 2]. Finally, a gingival morphology [5] and gingival phenotype, i.e., gingival thickness and keratinized tissue width, that can be altered with teeth malposition [6, 7] should be evaluated as a part of periodontal phenotype evaluation also including an evaluation of bone morphotype, i.e., buccal bone thickness. A wide variety of novel imaging methods are constantly being proposed to aid in the diagnosis of periodontal conditions, including ultrasound imaging [8], magnetic resonance imaging [9], cone-beam computed tomography [10, 11], optical coherence tomography [12], and optical scanning [13]. Recently, intraoral scans were utilized for the remote diagnosis of gingival and periodontal conditions by visual assessment [14, 15]. Both studies reported promising results regarding time efficiency and accuracy of the remote diagnosis that could be further increased with future technology improvements such as artificial intelligence [16].

In periodontal research, optical scanning enabled novel and accurate evaluation methods, such as volumetric assessment of soft tissue dynamics [17–19]. Recently, computer-aided analysis of optical scans revealed considerably improved accuracy of linear measurements of dimensional differences of soft tissues after different treatment procedures compared to evaluation with periodontal probe [20–23]. Furthermore, optical scans also present a possibility for shape analysis, successfully implemented in medical image analysis to aid in detecting and diagnosing [24–26]. Recently, palatal morphology was evaluated and compared, enabling twin differentiation or human identification due to the high accuracy of the intraoral optical scanning [27]. A different approach with surface curvature estimation was utilised in a validation study using intraoral optical scans to document palatal soft tissue shape and surface irregularity as a possible screening and diagnostic tool for oral cancer [28]. Furthermore, as visual assessment of gingival inflammation is based on the morphological changes of the gingival tissues [2, 4], it seems that evaluation of such changes by shape analysis might facilitate the detection of subtle changes associated with the presence, progression, and resolution of gingival conditions. Therefore, in both clinical and research settings, there is a need for the non-invasive, objective, and precise evaluation of gingival tissues and their changes.

Therefore, the present study aimed to propose an objective evaluation method for gingival shape analysis using surface curvature estimation of the intraoral optical scans by applying different curvature measures computed at various parameters on central and interdental gingiva.

## Methods

### Gingival shape analysis

The proposed method consisted of four main steps, i.e., image acquisition—optical scanning, surface curvature estimation, region of interest (ROI) definition, and gingival shape analysis.

#### Acquisition—optical scanning

The inclusion criterion was the presence of advanced periodontitis (Stage III, Grade B/C; [29]) with at least nine teeth (excluding molars) in the upper jaw. The exclusion criteria were periodontal treatment in the last six months, antibiotic treatment in the previous six months, chronic systemic diseases, and medication with known influence on periodontium or wound healing. Sixteen maxillary digital models, i.e., two per patient, were acquired in a scan-rescan manner using intraoral optical scanning (CEREC Omnicam AC, Dentsply Sirona; software version: SW 4.5.2). An experienced operator performed two consecutive scans of each patient to obtain the morphology of the tissues without any patient’s or operator’s actions in between the scans. Teeth from right to left premolars were included in the analysis resulting in a dataset of 78 maxillary teeth. One patient was additionally scanned 3-months after the non-surgical treatment of periodontitis to demonstrate the proposed method’s applicability. Digital models were exported in Standard Tessellation Language (STL) file format.

#### Surface curvature estimation

**Fig. 1:**
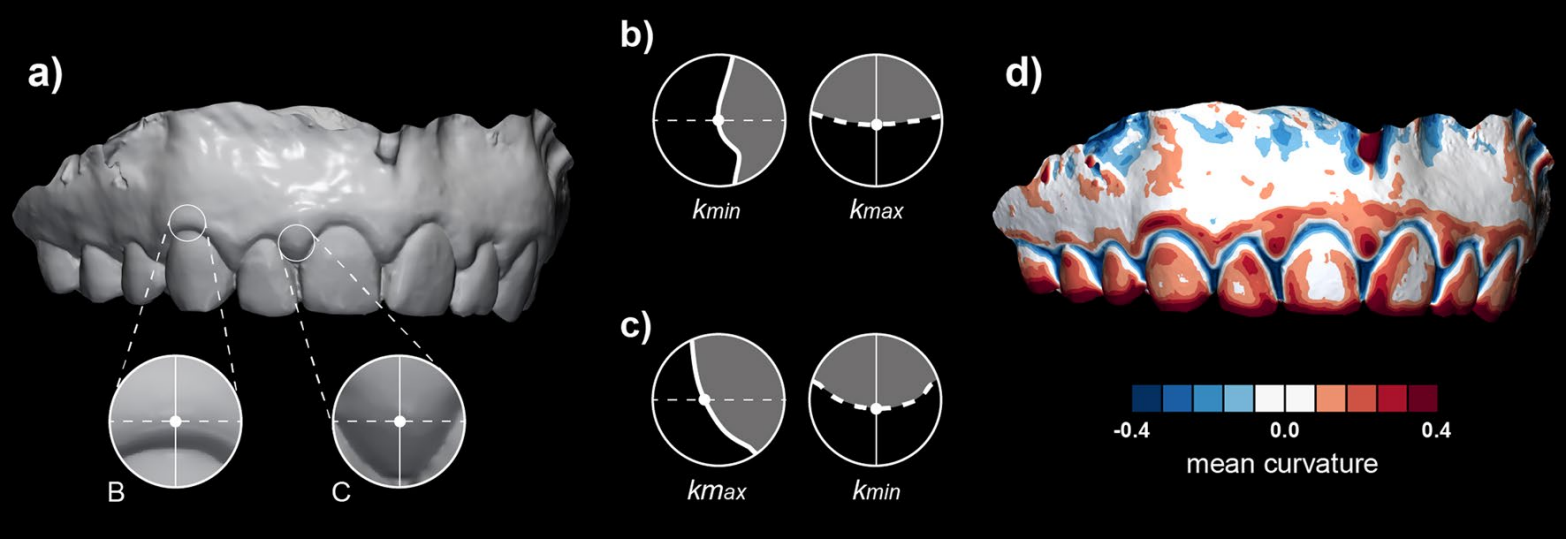
Concept of surface curvature estimation displayed on an intraoral scan (**a**). With a surface curvature estimation algorithm, principal curvature directions and values, i.e., maximum (k_max_) and minimum (k_min_), were computed for each point of the intraoral scan. Principal curvature directions for a selected point (i.e., the intersection of the white lines) of two anatomically different regions, i.e., central (B) and interdental (C), were displayed with corresponding cross-sections (**b** and **c**). The principal curvature directions coincide with corono-apical (full white line) and mesio-distal (dashed white line) directions in those two selected regions. Note that principal curvature directions are switched in central and interdental regions. The curvature value of each point’s surface is visualised with colour mapping (**d**)

Surface curvature is the quantity that measures how much the surface locally at each point deviates from the plane orthogonal to the surface at that point (called the tangent plane). The amount of deviation depends on the direction we choose in the tangent plane. The two directions where the amount of change is minimum and maximum are called principal curvature directions, and they correspond to the principal curvatures, i.e., maximum curvature k_max_ and minimum curvature k_min_ (Fig. 1). These two principal curvatures are eigenvalues of the shape operator [30] and can be combined in several ways to obtain a quantity representing a curvature measure [31], e.g.,

$$mean\;curvature(MC):H = \frac{{k_{{max}} + k_{{min}} }}{2}$$

$${\textit{Gaussian curvature (GC): K}} = {k}_{max}*{ k}_{min}$$

$${\textit{shape index (SI)}} = \frac{2}{\pi }arc\text{tan}\left(\frac{{k}_{min}+{k}_{max}}{{k}_{min}-{k}_{max}}\right)=\frac{2}{\pi }arc\text{tan}\left(\frac{-H}{\sqrt{{H}^{2}-K}}\right)$$

$${\textit{curvedness (CU)}} = \sqrt{\frac{{k}_{max}^{2}+{k}_{min}^{2} }{2} } = \sqrt{{2 H}^{2}-K}$$

Since in our case the scanned surface is not given as a smooth parametric surface, but just as a discrete triangular surface mesh, approximation algorithms must be used to estimate these quantities at each of the vertex of the triangular mesh. From the above formulas it is enough to estimate only the mean (MC) and Gaussian curvature (GC). The efficient and fast numerical algorithms are local, meaning that only the information of the position of the vertex together with positions of neighbouring vertices (chosen by a prescribed diameter) is used to compute MC and GC at each vertex. These algorithms are usually based on local least square bivariate polynomial or rational approximants [32] or the approximation of the Laplace-Beltrami operator using spatial averages [33].

**Fig. 2:**
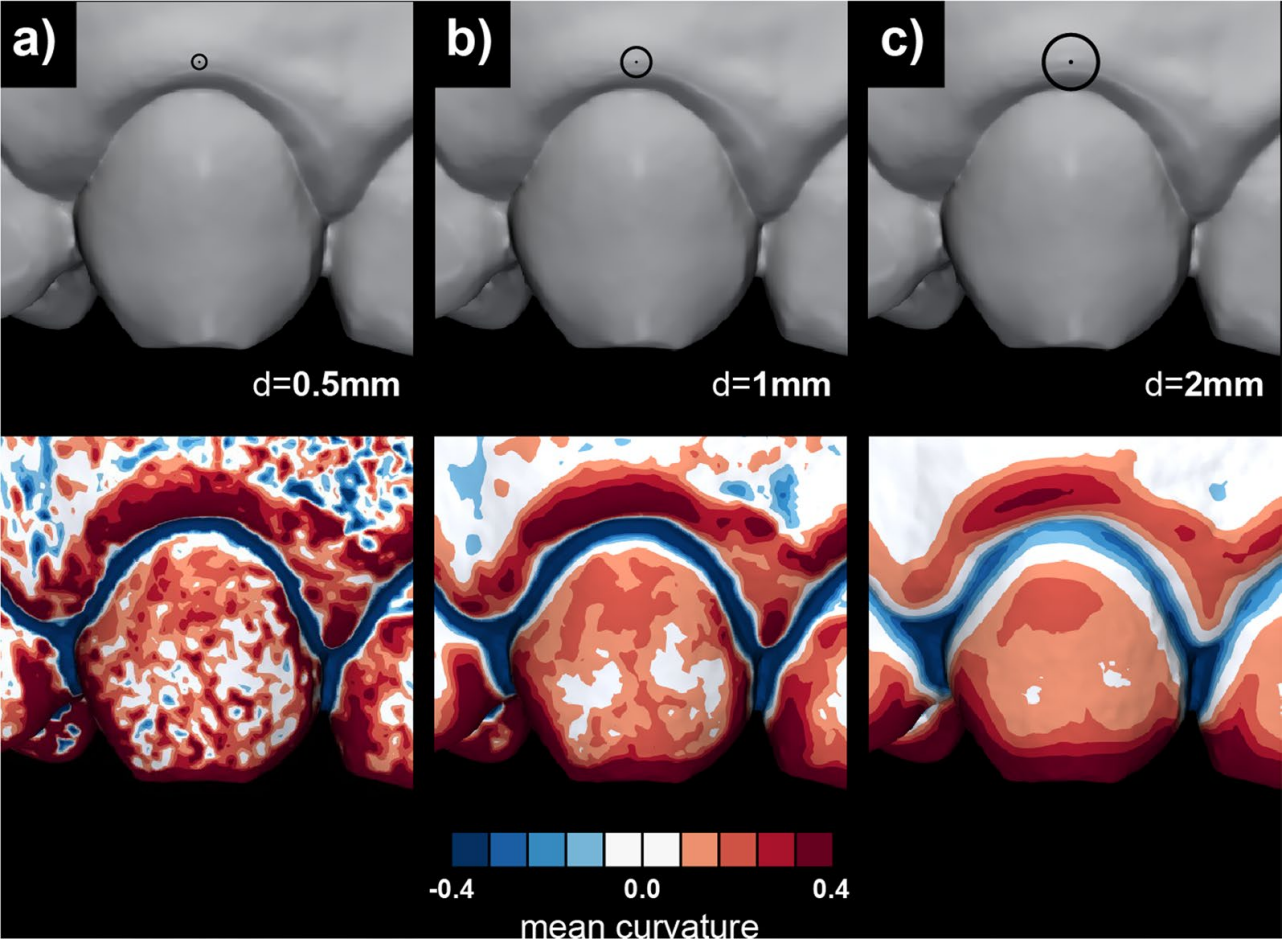
The effect of different diameter selection, ranging from 0.5 (**a**) to 2 mm (**c**), for surface approximation that gives surface curvature estimation. The diameter represents a threshold for detecting intraoral scans’ geometrical features

For our problem the surface curvature was computed using PyMeshLab, i.e., a Python library that interfaces to MeshLab (version 2021.10). A “Scale Dependent Quadric Fitting” curvature estimation method was used, enabling the definition of the diameter of interest. The diameter defines the neighbouring points of each vertex that are used to construct the approximating quadric (rational quadratic) surface, which then allows the computation of curvature measures at the vertex of interest. All curvature measures mentioned were computed at three different diameters, i.e., 0.5, 1, and 2 mm (Fig. 2).

Each model was exported in polygon file format (PLY), enabling the model to save computed curvature values for each point as a scalar value.

#### ROI definition

**Fig. 3:**
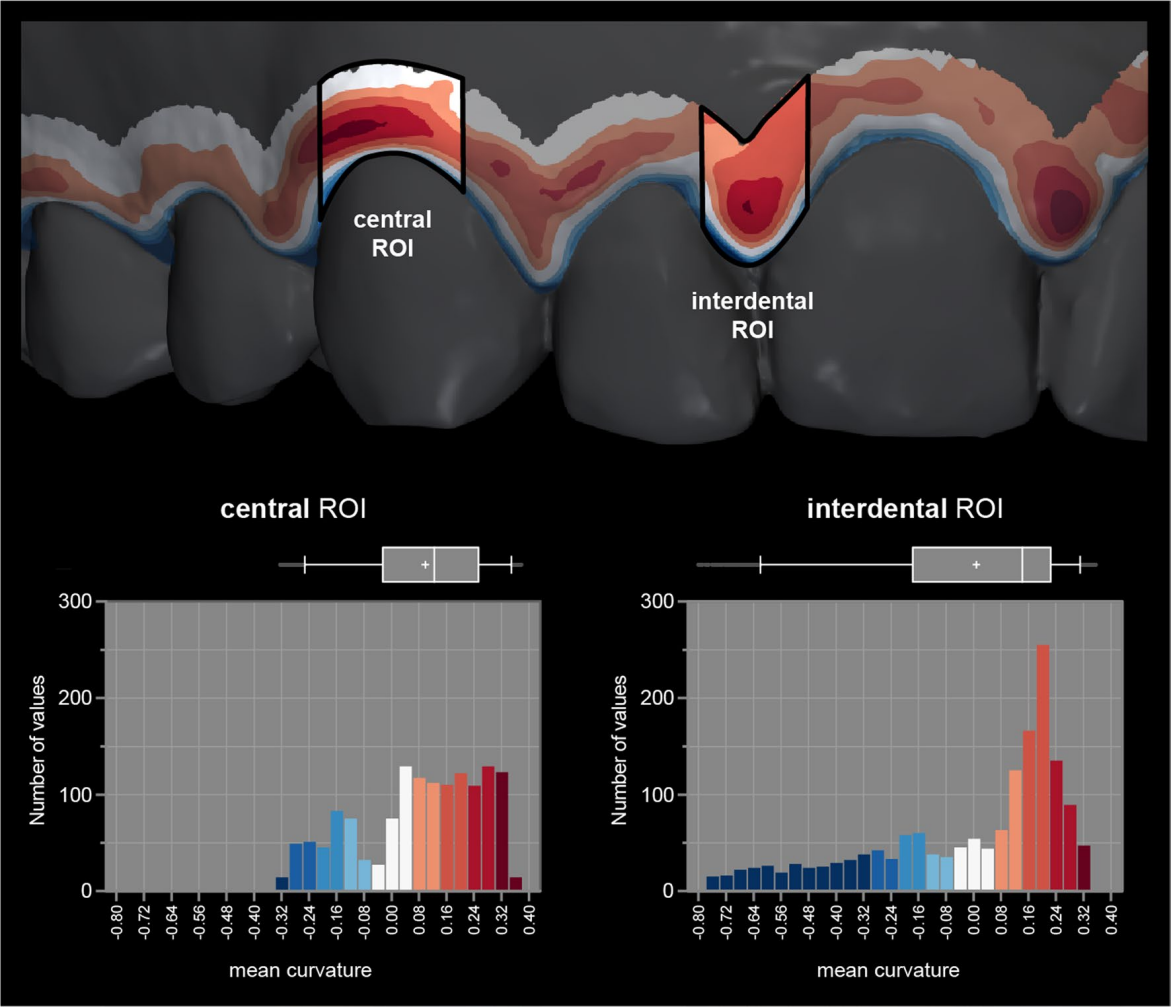
Two regions of interest (ROI), i.e., central and interdental, are displayed on a gingival margin band to facilitate focused visualisation. Corresponding histograms and boxplots are shown to display the distribution and variability of the curvature values. Local maximum values (red) are of interest for gingival shape analysis. Whiskers represent 5th and 95th percentile values, and + represents mean value

Two ROIs were defined on the gingival margin, i.e., central and interdental (Fig. 3). The ROI was defined with the aid of landmark curves in GOM Inspect (version 2018, GOM GmbH) and 3D Slicer (version 4.11.20210226) [34]. First, the gingival margin was defined with the method proposed by Kuralt et al. [35] utilizing surface curvature. Then, the gingival margin landmark curve was projected in the apical direction on the model’s surface with an offset of 3 mm, thus, limiting the ROI in the apical direction. Next, the tooth’s mesial and distal line angles were used to create cross-sections in the coronal-apical direction for mesial and distal limitations, delimiting central from interdental ROI. Finally, defined curves were exported in comma-separated values (CSV) file format.

#### Gingival shape analysis and visualisation

Scans with computed curvature values and landmark curves for ROI definition were imported into the Cloud Compare (version 2.12 alpha) for further analysis. All available scans with different curvature values per case were combined into one scan with multiple scalar fields. Thus, enabling standardised ROI selection using the Segment tool. Each ROI’s curvature value, i.e., 18 different combinations—six different curvature measures and three diameters, were exported to Excel (Microsoft 365, Microsoft) to calculate descriptive statistics. The distribution of curvature values within the ROI depended on the ROI’s size and included geometrical features. The selected ROI also included a small part of the gingival margin (Fig. 3—blue) that shifted the mean values per ROI towards zero (Fig. 3—boxplots and histograms). To avoid this effect, the 95th percentile of the curvature values of each ROI and curvature measure was used to measure the local maximum shape of the gingiva (gingival shape parameter—GS).

Curvature values were visualised only at the gingival margin with a discrete threshold colour scale [36] to facilitate the observation of gingival shape (Fig. 3).

### Method validation

The reproducibility of surface curvature estimation for gingival shape analysis was evaluated by computing the surface curvature on two consecutive scans acquired at the same time-point with a scan-rescan process. The mean of the absolute differences in the GS, i.e., the 95th percentile of the curvature values in the ROI, was used as a methodological error between scan and rescan at each diameter and curvature measure.

## Results

### Validation of the proposed method

**Fig. 4:**
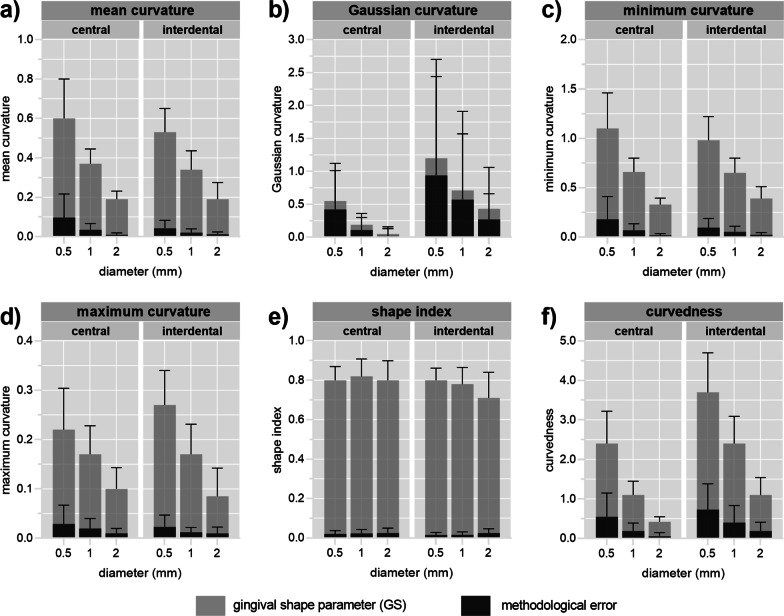
Mean and standard deviation of gingival shape parameter (GS) (i.e., the 95th percentile of the curvature values)—light grey, and method error, i.e., the absolute difference in the GS between scan and rescan—dark grey, obtained for each diameter and curvature measure for a study sample represented with bar charts. The central and interdental region sample size was 78 and 88, respectively

The curvature values and methodological error of the central and interdental ROI for each curvature measure and diameter are displayed with bar charts similar to signal-to-noise charts.

(Fig. 4). The mean GS of a study sample (Fig. 4—light grey) decreases in all curvature measures with increasing diameter except for the SI, indicating that all measures except SI are size-dependent (Fig. 4e). Visual inspection of colour-coded curvature maps also confirms a decrease of curvature values with increasing diameter exhibited by the decreased intensity of colours (Fig. 2).

The methodological error, i.e., absolute differences in the GS between scan and rescan values (Fig. 4—dark grey), also decreases with an increasing diameter (Fig. 4). The methodological error represents a minor part in relation to the GS except for the GC and CU measures (Fig. 4b and f).

For central ROI, k_min_ measure computed at 2 mm diameter seems optimal parameter to quantify shape with mean (and standard deviation) of 0.33 (0.07) for a study sample. While MC measure computed at 2 mm diameter seems optimal parameter to quantify shape for interdental ROI with mean (and standard deviation) of 0.19 (0.09) for a study sample.

### Demonstration of the proposed method

**Fig. 5:**
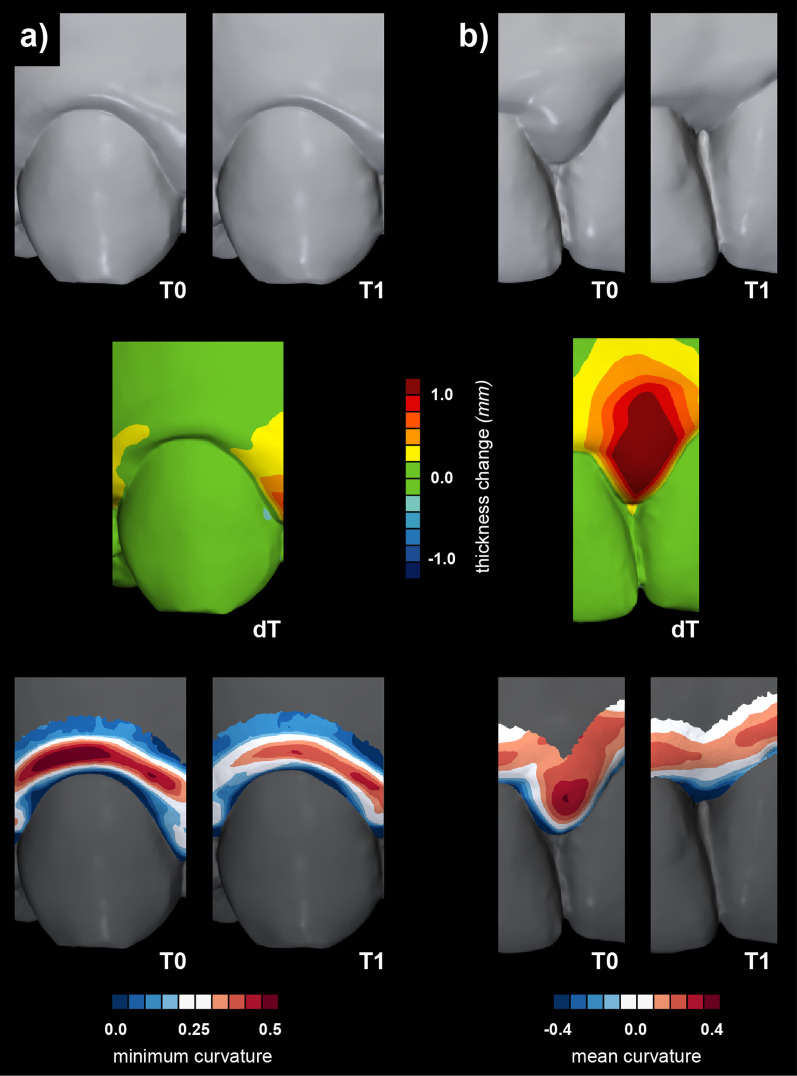
The ability of the novel method for gingival shape analysis using surface curvature to detect subtle differences in soft tissue dynamics undetectable by volumetric assessment. Baseline (T0) and three-month follow-up (T1) intraoral scans associated with the non-surgical periodontal treatment (upper row) of two anatomically different regions, i.e., central (**a**) and interdental (**b**). The scans were superimposed and evaluated in terms of tissue thickness changes (middle row) and with a novel gingival shape analysis (bottom row). Changes in the interdental regions are already apparent when comparing the scans side by side and even more pronounced with the volumetric colour-coded map. Changes are also confirmed with the gingival shape analysis using mean curvature measure (**b**). It should be outlined that the region for gingival shape analysis also shifted in the apical direction with the loss of the tissues. However, the regions were defined wide enough to capture the local maximum. In contrast, changes in central regions are subtle and barely visible with direct observation but are demonstrated by gingival shape analysis using minimum curvature measure (**a**)

**Fig. 6:**
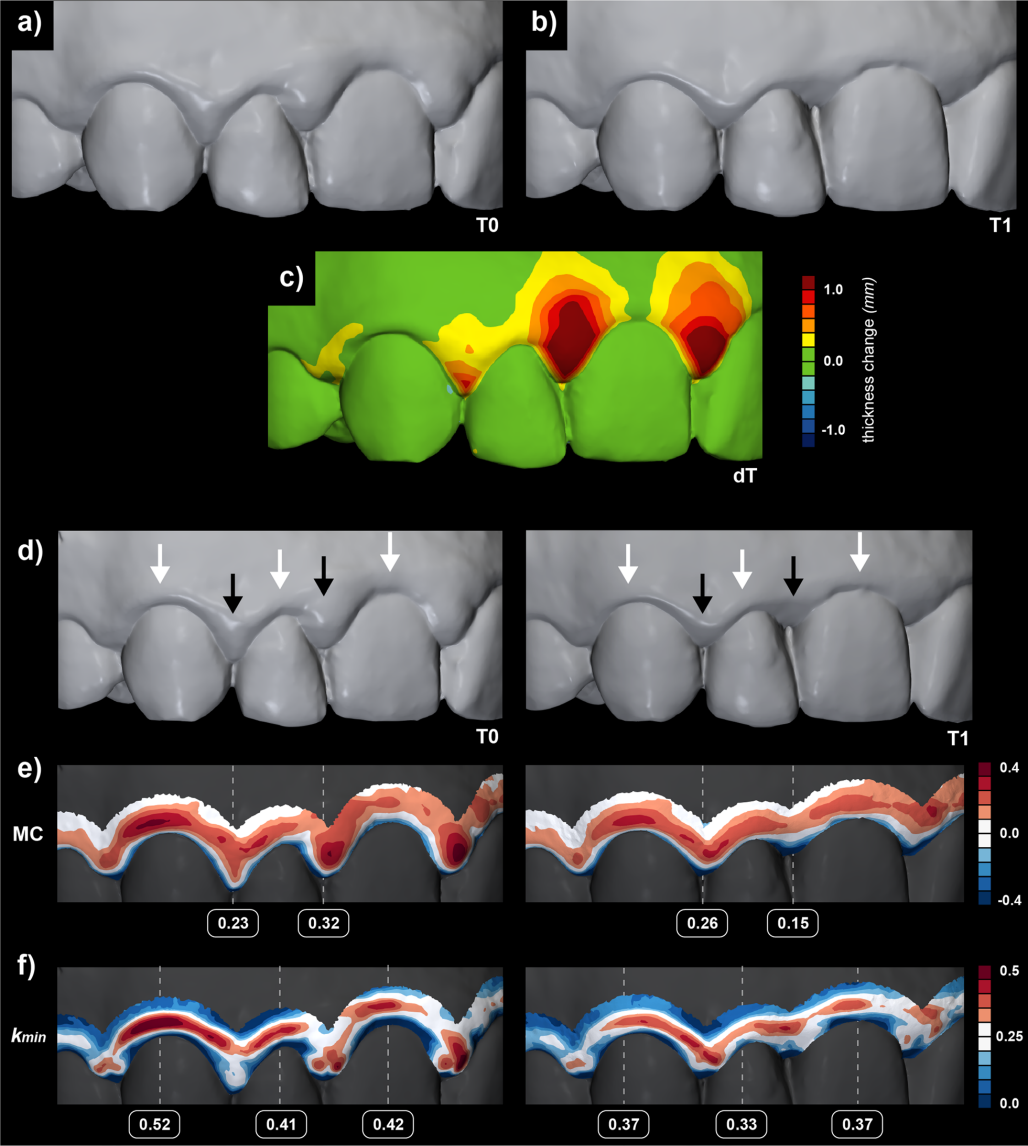
Evaluation of soft tissue dynamics associated with periodontitis treatment by volumetric assessment (**a**—baseline scan (T0), **b**—follow-up scan (T1), and **c**—colour-coded map) and a novel gingival shape analysis using surface curvature (**d**—T0 and T1 scans, **e**—mean curvature (MC) and **f**—minimum principal curvature(k_min_)). Five different regions were selected based on the colour-coded map (**c**), i.e., two interdental with loss of the tissues above 1.0 mm (black arrows) and three central with changes below the 0.4 mm threshold (white arrows) (**d**)

The proposed method for gingival shape analysis using surface curvature estimation was demonstrated in a clinical case of periodontitis treatment (Figs. 5 and 6). Furthermore, visual observation of the cross-sections displayed in Fig. 1 and colour-coded models with all curvature measures computed at 2 mm diameter (Additional file 1: Fig. 1) confirmed that k_min_ and MC measures seem optimal parameters to quantify shape at central and interdental ROI, respectively.

## Discussion

The surface curvature estimation of the intraoral scans presents a precise method for gingival shape analysis demonstrated by a high ratio of curvature values versus methodological error. Gingival tissues’ shape seems to be optimally evaluated by the k_min_ for central and MC for interdental region, both computed at 2 mm diameter. Objectively describing and quantifying the gingival tissues’ shape may aid in evaluating morphological variability, detecting and diagnosing early periodontal conditions, and may play an important role in following changes over time.

To the best of our knowledge, this is the first study attempting to use non-invasive intraoral scans for quantitative evaluation of the gingiva in terms of shape. Many non-invasive digital methods were proposed to overcome the subjectivity of gingival assessment [4]. Those methods are based either on colour changes, i.e., redness [37, 38], or volumetric changes of the tissues, i.e., oedema [13, 39, 40], with the latter indirectly evaluating shape through dimensional differences. In medicine, shape analysis is a well-established approach utilizing various methods [41]. In periodontology, digital evaluation predominantly means just the digitalization of the established evaluation methods [42]. Only recently, curvature analysis was used for segmentation of teeth [43–45] and to detect anatomical landmarks, i.e., cemento-enamel junction and gingival margin, to automate gingival recession measurements [46] and evaluate changes over time [20, 35]. Surface curvature estimation utilised for the palatal soft tissue shape quantification [28] and the results of the present study indicate that surface curvature estimation of optical scans presents a suitable method for soft tissue shape analysis. The proposed method facilitates detecting subtle changes of the tissues almost not detected by existing methods as observed in colour-coded distance maps (Figs. 5 and 6), proposing complimentary use of the existing and proposed methods. Differences could also be evaluated in baseline gingival shape concerning craniofacial characteristics and periodontal phenotype.

Definition of the diameter for the surface approximation in the surface curvature estimation is essential for optimal detection of gingival shape. Therefore, local geometric analysis often requires surface modification, i.e., smoothing or surface approximation with a defined diameter of interest prior to the computation [47]. Such surface modification or approximation aims to preserve relevant features and suppress noise and details below a certain threshold of interest for analysis, i.e., the defined diameter. The present study used a local size-dependent curvature estimation method with the diameters defined through empirical observation. The results revealed that robustness to noise increases with an increasing diameter which is also evident with a decrease in methodological error (Figs. 2 and 4). According to the results, a diameter of 2 mm yielded the most relevant colour-coded curvature maps displaying morphologically relevant features (Fig. 2).

Central and interdental gingiva are two distinct regions regarding their shape, affecting the selection of optimal curvature measures. The shape of the central gingiva mainly depends on the tooth’s position in the dental arch, the position of the root in relation to the alveolar process, and the anatomy of the root [48]. Those relations are typically displayed as a mesial-distal convexity of central gingival as observed with a mesial-distal cross-section of a maxillary canine (Fig. 1b) and colour-coded curvature maps using k_max_ (Additional file 1: Fig. 1). In the absence of pathology, the central gingiva is tightly adapted to the underlying hard tissues with a knife-edge margin, i.e., observing the coronal-apical profile [48]. With gingival inflammation, swelling occurs, additionally and reliably displayed with k_min_ (Figs. 1b and 5a). In contrast, the shape of the interdental gingiva is more complex. It depends on the contour of the proximal tooth surfaces, underlying bone support, and gingival embrasures’ size, shape, and location [48]. As the bone support of two neighbouring teeth may differ, interdental ROI was not further divided into mesial and distal ROI. Observing cross-sections at the interdental gingiva revealed that principal curvatures are contrarywise as with central gingiva and are more similar regarding values (Fig. 1b and **c).** Therefore, combining both principal curvatures, such as MC, seems better to describe the swollen interdental gingiva (Fig. 5b). However, the analysis with its parameters and clinical relevance needs to be further tested for specific clinical scenarios and datasets to enable practical quantitative gingival shape analysis using optical scans.

For effective implementation of a novel method, comprehensive yet straightforward evaluation and visualisation of all available data is essential. Cross-section image evaluation, which is most extensively used in periodontology and implantology, represents only one of the numerous possible sections that are not being evaluated and displayed. More specifically, the curvature is measured only in the direction of the cross-section (Fig. 1) [46]. In comparison, the 3D approach for evaluating and visualising shape with surface curvature has a significant advantage because it allows measurement of the curvature independent of the specific locations and orientations of imaging. Furthermore, surface curvature analysis is an excellent example of overcoming evaluation with several standardised yet arbitrary preselected measuring sites, potentially omitting relevant information [49]. Such a comprehensive approach may also represent a considerable step toward personalized or precision dentistry [50, 51].

The benefit of intraoral scanning as a diagnostic tool may present an added value to the clinical workflow either in terms of evaluation of disease presence or progression [52, 53]. Despite significant investment in money, intraoral scanners are increasingly present and used in dental practices [54]. Digital models obtained with optical scanning can be magnified and viewed from different directions, facilitating detailed inspection of the models due to the high resolution [55]. Utilization of intraoral scanning for objective evaluation possibilities, such as follow-up with superimposition of the scans and shape analysis, would enhance a conventional clinical examination and increase early detection. The importance of early detection and prevention of gingivitis as a first step toward preventing periodontitis [56, 57] was also recently emphasized by a recent report on periodontitis’ financial and human costs commissioned by the [58].

The present study was subjected to some limitations. First, the proposed method requires specific computer knowledge with 3D analysis software experience. Therefore, open-source software for processing and editing 3D digital models, i.e., MeshLab, was utilised as the leading intuitive and easy software. Furthermore, due to the combination of different settings, many required computations were efficiently performed using basic programming to batch the process. Second, other intraoral scanners should be tested as well. However, regarding surface curvature estimation, the effect of the initial scan, i.e., surface mesh composition, is minimised due to selecting the diameter of interest. Third, selecting a discrete colour scale requires a definition of arbitrarily defined thresholds that may not be yet settled from the perspective of clinical relevance. Further studies are required correlating existing knowledge to define such thresholds.

## Conclusions

Within the limitations of the present study, surface curvature estimation of the intraoral scans seems to be a precise and reproducible method for gingival shape analysis. However, further studies are required to correlate shape in terms of morphological variability and presence of inflammation with clinical data.

## Supplementary Information

**Additional file 1: Supplementary Figure 1.** Baseline (T0) and three-month follow-up (T1) after non-surgical periodontal treatment intraoral scans (upper row) were evaluated with gingival shape analysis using surface curvature using available curvature measures. Five different regions were selected to outline the gingival shape changes, i.e., two interdental with loss of the tissues above 1.0 mm (black arrows) and three central with changes below the 0.4 mm threshold (white arrows).

## Data Availability

The data that support the findings of this study are available on a reasonable request from the corresponding author. The data are not publicly available due to privacy or ethical restrictions.

## Abbreviations

CSV: Comma-separated values
CU: Curvedness
GC: Gaussian curvature
GS: Gingival shape parameter
K_max_: Maximum curvature
k_min_: Minimum curvature
MC: Mean curvature
PLY: Polygon file format
ROI: Region of interest
SI: Shape index
STL: Standard Tessellation Language
