# Correction: Gingival shape analysis using surface curvature estimation of the intraoral scans

**DOI:** 10.1186/s12903-022-02554-y

**Published:** 2022-12-09

**Authors:** Marko Kuralt, Alja Cmok Kučič, Rok Gašperšič, Jan Grošelj, Marjeta Knez, Aleš Fidler

**Affiliations:** 1 Department of Restorative Dentistry and Endodontics, University Medical Centre Ljubljana, Hrvatski trg 6, 1000 Ljubljana, Slovenia; 2 Faculty of Medicine, University of Ljubljana, Ljubljana, Slovenia; 3 Public Health Centre Celje, Celje, Slovenia; 4 Department of Oral Medicine and Periodontology, University Medical Centre Ljubljana, Ljubljana, Slovenia; 5 Department of Oral Medicine and Periodontology, Faculty of Medicine, University of Ljubljana, Ljubljana, Slovenia; 6 Faculty of Mathematics and Physics, University of Ljubljana, Ljubljana, Slovenia; 7 Department of Endodontics and Operative Dentistry, Faculty of Medicine, University of Ljubljana, Ljubljana, Slovenia

**Correction: BMC Oral Health 22, 283 (2022)**

**https://doi.org/10.1186/s12903-022-02322-y**

Following the original article's publication [1], the authors identified a terminology issue regarding the principal curvatures, i.e., maximum (k_max_) and minimum (k_min_), and maximum (Max) and minimum (Min) curvature measures computed and used in the present study. The algorithm used in the study (i.e., Scale Dependent Quadric Fitting) as implemented in PyMeshLab interfacing to MeshLab (version 2021.10) uses the terms »Max Curvature« (Max) for k_min_ and »Min Curvature« (Min) for k_max_ and may therefore confuse the reader.

Following corrections were made for clarification of above issue:

- **Figure 1:** k_min_ and k_max_ were interchanged in b) and c). Revised figure:
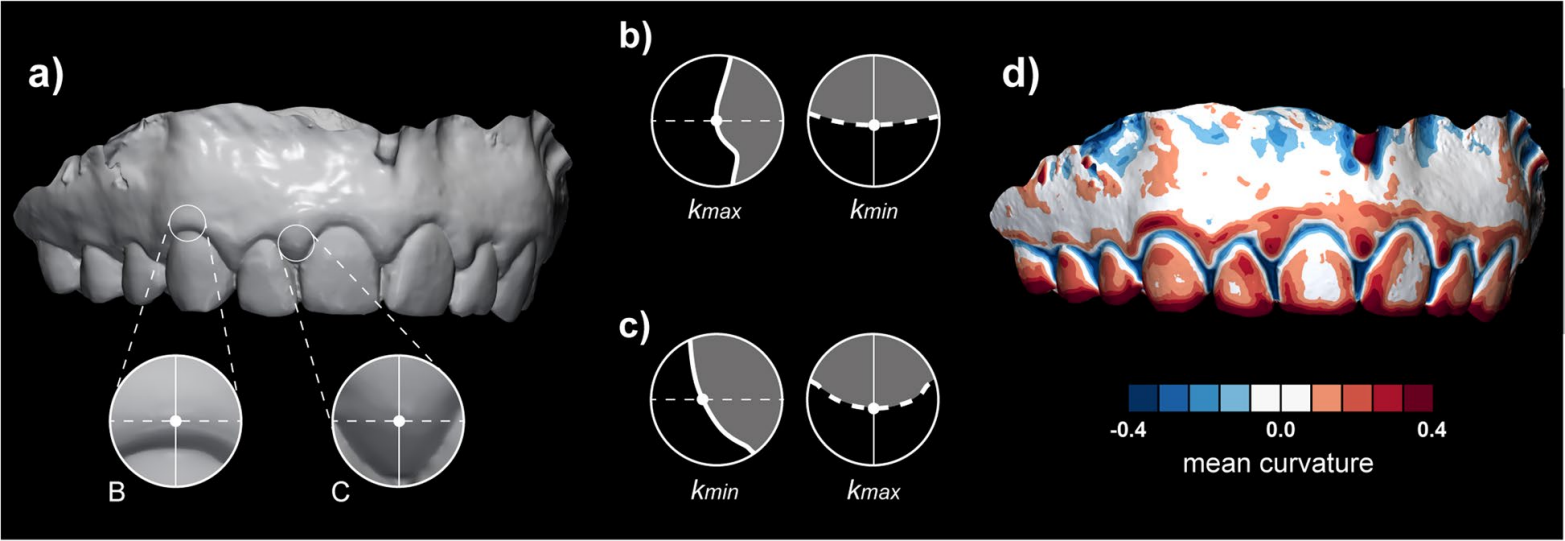

- **Methods – Surface curvature estimation** (pg. 3): Following sentence was revised: »All curvature measures mentioned above, i.e., MC, GC, SI, CU, and minimum (Min) and maximum curvature (Max) as implemented in the software were computed at three different diameters, i.e., 0.5, 1, and 2 mm (Fig. 2).«
- **Results - Validation of the proposed method** (pg. 4): Following sentence was revised (k_min_ interchanged to Min): »For central ROI, Min measure computed at 2 mm diameter seems optimal parameter to quantify shape with mean (and standard deviation) of 0.33 (0.07) for a study sample.«
- **Results - Demonstration of the proposed method** (pg. 5): Following sentence was revised (k_min_ interchanged to Min): » Furthermore, visual observation of the cross-sections displayed in Fig. 1 and colour-coded models with all curvature measures computed at 2 mm diameter (Additional file 1: Fig. 1) confirmed that Min and MC measures seem optimal parameters to quantify shape at central and interdental ROI, respectively.«
- **Discussion** (pg. 5): Following sentence was revised (k_min_ interchanged to Min): »Gingival tissues’ shape seems to be optimally evaluated by the Min for central and MC for interdental region, both computed at 2 mm diameter.«
- **Figure 6:** k_min_ was changed into Min in f)
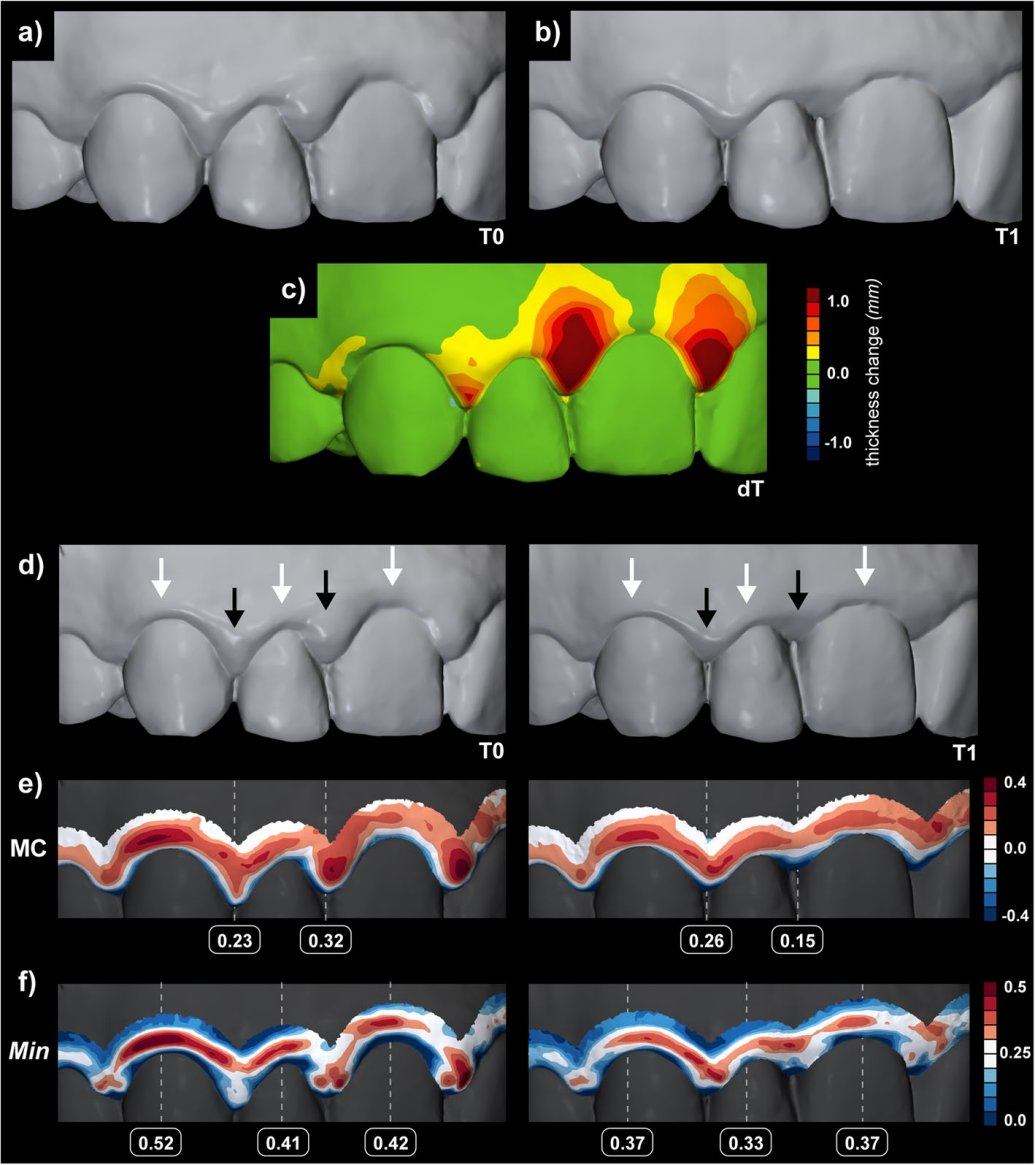

- **Discussion** (pg. 8): Following sentence was revised (k_max_ interchanged to Max): »Those relations are typically displayed as a mesial-distal convexity of central gingival as observed with a mesial-distal cross-section of a maxillary canine (Fig. 1b) and colour-coded curvature maps using Max (Additional file 1: Fig. 1).
- **Discussion** (pg. 9): Following sentence was revised (k_min_ interchanged to Min): »With gingival inflammation, swelling occurs, additionally and reliably displayed with Min (Fig. 5a).«
- **Abbreviations** (pg. 9): The difference was outlined with revising and adding following abbreviations: »k_max_: Maximum principal curvature; k_min_: Minimum principal curvature; Max: maximum curvature measure; Min: minimum curvature measure«

## References

[1] Kuralt M (2022). Gingival shape analysis using surface curvature estimation of the intraoral scans. BMC Oral Health..

