# Supplementary material for: Gingival shape analysis using surface curvature estimation of the intraoral scans
Source: BMC Oral Health. 2022 Jul 12;22:283. doi: 10.1186/s12903-022-02322-y (PMC9275066; doi:10.1186/s12903-022-02322-y)
Supplement: Supplementary file 1 — Additional file 1: Supplementary Figure 1. Baseline (T0) and three-month follow-up (T1) after non-surgical periodontal treatment intraoral scans (upper row) were evaluated with gingival shape analysis using surface curvature using available curvature measures. Five different regions were selected to outline the gingival shape changes, i.e., two interdental with loss of the tissues above 1.0 mm (black arrows) and three central with changes below the 0.4 mm threshold (white arrows). [file 12903_2022_2322_MOESM1_ESM.docx]

**Supplementary material**

**
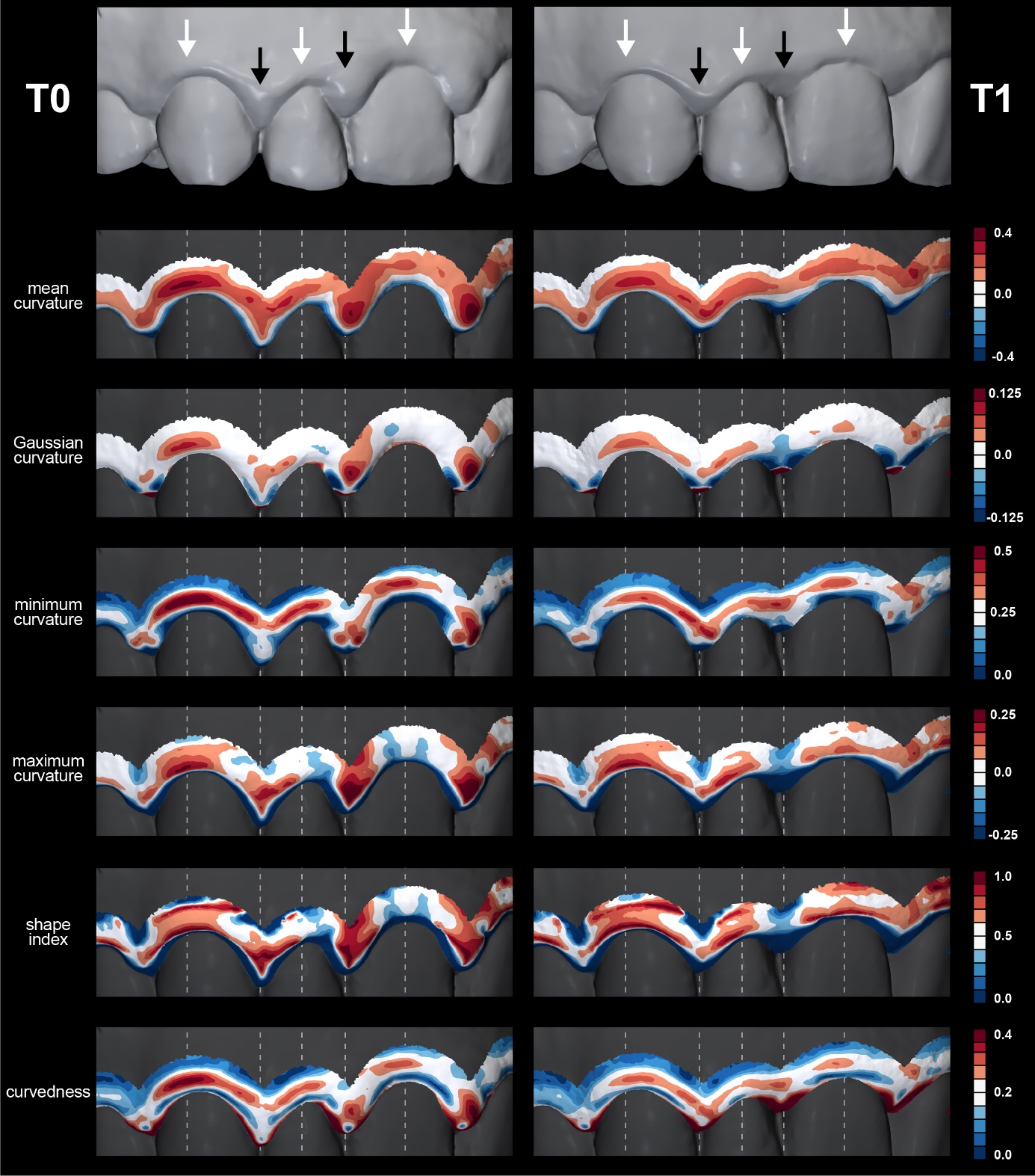
**

**Supplementary Figure 1.** Baseline (T0) and three-month follow-up (T1) after non-surgical periodontal treatment intraoral scans (upper row) were evaluated with gingival shape analysis using surface curvature using available curvature measures. Five different regions were selected to outline the gingival shape changes, i.e., two interdental with loss of the tissues above 1.0 mm (black arrows) and three central with changes below the 0.4 mm threshold (white arrows).
